# Gray Matter Volume and Cognitive Performance During Normal Aging. A Voxel-Based Morphometry Study

**DOI:** 10.3389/fnagi.2018.00235

**Published:** 2018-08-03

**Authors:** Stephen Ramanoël, Elena Hoyau, Louise Kauffmann, Félix Renard, Cédric Pichat, Naïla Boudiaf, Alexandre Krainik, Assia Jaillard, Monica Baciu

**Affiliations:** ^1^INSERM/CNRS, Institut Vision, Sorbonne University, Pierre and Marie Curie Universities (UPMC) Paris 06, Paris, France; ^2^CNRS LPNC UMR 5105, University of Grenoble Alpes, Grenoble, France; ^3^CNRS, Grenoble INP, GIPSA-lab, University of Grenoble Alpes, Grenoble, France; ^4^UMS IRMaGe Grenoble Hospital, University of Grenoble Alpes, Grenoble, France; ^5^Grenoble Institute of Neuroscience, University of Grenoble Alpes, Grenoble, France

**Keywords:** aging, gray matter, MRI, VBM, brain, cognitive

## Abstract

Normal aging is characterized by decline in cognitive functioning in conjunction with extensive gray matter (GM) atrophy. A first aim of this study was to determine GM volume differences related to aging by comparing two groups of participants, middle-aged group (MAG, mean age 41 years, *N* = 16) and older adults (OG, mean age 71 years, *N* = 14) who underwent an magnetic resonance images (MRI) voxel-based morphometry (VBM) evaluation. The VBM analyses included two optimized pipelines, for the cortex and for the cerebellum. Participants were also evaluated on a wide range of cognitive tests assessing both domain-general and language-specific processes, in order to examine how GM volume differences between OG and MAG relate to cognitive performance. Our results show smaller bilateral GM volume in the OG relative to the MAG, in several cerebral and right cerebellar regions involved in language and executive functions. Importantly, our results also revealed smaller GM volume in the right cerebellum in OG relative to MAG, supporting the idea of a complex cognitive role for this structure. This study provides a broad picture of cerebral, but also cerebellar and cognitive changes associated with normal aging.

## Introduction

Normal aging is associated with various behavioral, cognitive and cerebral changes that affect multiple functions such as attention (Geerligs et al., 2014), working memory (Nyberg et al., 2012), processing speed (Seidler et al., 2010) and cognitive control (Coppin et al., 2006; Salthouse, 2010; Marchand et al., 2011). The degree of cognitive decline is not identical for all cognitive processes, i.e., some functions undergo more severe change than others. For example, while executive functions are typically the first to show impairment during normal aging, language abilities remain relatively intact (Meyer and Federmeier, 2010), or even improve in terms of vocabulary, semantics and speech processing (Kavé et al., 2009; Verhaegen and Poncelet, 2013; cited in Baciu et al., 2016). Cognitive abilities can be generally classified as domain-general (e.g., executive functioning) and domain-specific functions (e.g., language abilities; Fedorenko, 2014; Karmiloff-Smith, 2015; Karmiloff-Smith et al., 2016). A large body of evidence indicates that aging modifies the cerebral representation of domain-general and domain-specific cognitive processes in terms of functional reorganization. For instance, several studies have reported increased involvement of anterior frontal areas, relative to posterior occipital and medial temporal areas during tasks involving both domain-general and domain-specific cognitive processes associated with aging (Gutchess et al., 2005; Davis et al., 2008; see Wingfield and Grossman, 2006; Morcom and Johnson, 2015; Helder et al., 2016), suggesting supplemental recruitment of executive areas to perform a given task. The modulation of cerebral activity by performance is another important aging effect. Aging, however, is not a uniform process, old adults vary in the degree of their cognitive abilities’ decline. For example, Wierenga et al. (2008) showed that, in addition to the predominant left-hemispheric activation classically elicited during word retrieval, older adults tend to exhibit supplementary right hemispheric activation that is modulated by their performance: the higher the performance, the higher the activation in right inferior frontal cortex. Other studies also point to a dissociation between high- and low-performing older adults, with the latter exhibiting less hemispheric asymmetry reduction than the former (Cabeza et al., 2002).

In terms of brain anatomy, normal aging is associated with structural changes, on account of extensive gray matter (GM) atrophy (Raz et al., 2005; Driscoll et al., 2009). It is worth noting that, as is the case with cognitive functions, the spatial localization and degree of atrophy are not homogeneous across the brain in older adults (for a review see Fjell et al., 2014). The frontal and temporal lobes exhibit the highest degree of GM atrophy. Substantial changes have also been observed in the parietal lobe, whereas the occipital lobe appears to remain relatively intact. Recent findings have also reported GM volume reduction in the cerebellum (Good et al., 2001; Alexander et al., 2006; Abe et al., 2008) including a number of cerebellar regions that are mainly involved in cognitive rather than motor functions (Buckner, 2013).

In order to noninvasively assess the structural cerebral changes associated with normal aging *in vivo*, voxel-based morphometry (VBM) has now become a routine method in the neuroimaging community. The VBM method enables an automated, quantitative and objective evaluation of the tissue volume (GM volume) across the brain (Kurth et al., 2015). Cross-sectional and longitudinal studies have demonstrated clear relationships between cognitive decline and the atrophy of specific brain regions. For example, reduced performance in episodic memory (EM) in normal older adults is correlated with reduced volume of the entorhinal cortex of the medial temporal lobe (Jessen et al., 2006). Similarly, executive deficits in normal aging are associated with greater atrophy of prefrontal regions (Raz and Rodrigue, 2006). However, only a limited number of longitudinal studies have evaluated the link between cognition in general and GM (Nyberg et al., 2010; Lovden et al., 2013; Pudas et al., 2013; Gorbach et al., 2017; Leong et al., 2017) and only a few were language-specific (Shafto et al., 2010).

In the present study, we evaluated the effect of age (older group, OG relative to middle-aged group, MAG) on GM volume, using a whole brain voxel-related GM analysis, in conjunction with a cognitive-score evaluation. To this end, we focused on how GM volume differs between MAG and OG in domain-specific regions involved in language and semantic memory but also in the domain-general regions involved in transversal cognitive processes and executive functions (i.e., the high-level cognitive abilities necessary for successful adaptive behavior to cope with complex situations). Moreover, given the fact that cerebellar atrophy occurs with age (Good et al., 2001; Alexander et al., 2006; Abe et al., 2008) and as more and more studies have emphasized the important role of this structure for cognition (see for example Sokolov et al., 2017) we also included a specific pipeline for cerebellum analyses. In comparison with standard pipelines for whole brain analyses, the present one allowed us to improve the accuracy of inter-subject alignment during normalization step and removal of supra-tentorial GM which could bias final results. We carried out a whole-brain analysis and specific cerebellar analysis to evaluate GM differences between middle-aged and older adults relative to middle-aged adults, we expected old adults to exhibit smaller GM volume in cerebral and cerebellar regions involved in both domain-general and specific processes, such as frontal areas involved in language processes and executive functioning. Furthermore, we expected this reduced GM volume to be associated with differences in performance in cognitive tests evaluating the respective processes.

## Materials and Methods

### Participants

Among the 31 healthy participants initially included, we retained 30 who were divided into two age groups: a MAG (*N* = 16; 11 males; mean age ± standard deviation (SD): 40.8 ± 8.6 years; range 30–57 years, and an older group (OG, *N* = 14; 10 males; mean age ± SD: 70.5 ± 6.6 years; range 59–84). The excluded participant was 84 years old and showed aberrant (superior to 2*SD OG) GM (543 cm^3^) and cerebrospinal fluid (CSF) volume (706 cm^3^) values relative to the mean CSF and GM volumes of the OG (CSF; mean = 389 cm^3^; SD: 107 cm^3^; GM: mean = 664 cm^3^; SD: 57 cm^3^). All participants were native French speakers; had a high level of education (mean ± SD MAG: 4 ± 0; OG: 3.85 ± 0.36; low-performing OG (lOG): 3.85 ± 0.36; high-performing OG (hOG): 3.85 ± 0.36) as measured by the Poitrenaud questionnaire (Kalafat et al., 2003). There was no difference in education level between groups (MAG vs. OG and lOG vs. hOG). Participants were right-handed (Edinburgh Handedness Inventory; Oldfield, 1971); had normal or corrected-to-normal vision; and reported no history of neurological disorder or sensorimotor dysfunctions. The other inclusion criteria were the absence of general cognitive (Mini Mental State Examination, MMSE; Folstein et al., 1975) or EM (“5 words” test; Dubois et al., 2002) deficits, as well as the absence of anxiety and depression (Hospital Anxiety and Depression scale, HAD; Zigmond and Snaith, 1983). Participants gave their written informed consent to participate in the study which was approved by the local ethics committee (CPP N°: 2014-A00569-38; Comité de protection des personnes Sud Est (South East People’s Protection Committee)). The demographic and inclusion criteria are reported in Table 1.

**Table 1:** Demographic information and inclusion criteria for all participants.

Gender (M/F)	Groups					
	MAG 11/5		OG 10/4			
	Mean	SD	Mean	SD	*T* test	*P* value
Age	40.8	8.6	70.5	6.57	−10.5	<0.05*
Edinburgh Scale	86.3	15.9	90	11.87	1.578	0.126
ESC	4	0	3.85	0.36	−0.712	0.482
MMSE	29.3	1.5	29.07	1.2	0.352	0.728
HAD_A	6.3	2.6	6.21	1.96	0.117	0.908
HAD_D	2.8	2.5	3.78	2.93	−1.05	0.303
EM	9.94	0.25	9.92	0.26	0.095	0.925

*We performed two sample t-tests to compare the younger and older groups. Our analyses revealed that these two groups differed only in terms of age. MAG, Middle-aged group; OG, Older group; ESC, Education and Socio-cultural Level; MMSE, Mini-Mental State Evaluation; HAD_A, Hospital Anxiety scale; HAD_D, Hospital Depression scale; EM, Episodic Memory; SD, Standard Deviation. Bold values with an asterisk indicate significant differences*.

### Cognitive Assessment

Several cognitive tests were administered to evaluate the cognitive level of each participant in terms of domain-general and domain-specific processes. The domain-general evaluations included short-term memory (Digit Span Memory test; Weschler, 2008); processing speed and mental flexibility (Trail Making Test part B, TMT-B; Tombaugh, 2004); visual scanning and motor processes (Trail Making Test part A; TMT-A; Tombaugh, 2004); and a global evaluation of executive functions and frontal efficiency (Frontal Assessment Battery, FAB; Dartinet and Martinaud, 2005). Domain-specific evaluations concerned mainly language and semantic memory and included assessments of vocabulary and verbal intelligence (Mill-Hill vocabulary scale; Deltour, 1993); lexical retrieval and generation (picture naming, PN; DO-80, Metz-Lutz et al., 1991); semantic processing (Pyramid and Palm Tree test, PPT; Howard and Patterson, 1992) and the Verbal Automatisms test (Beauregard, 1971), which was used to determine levels of overlearned semantic information. As this test is sensitive to aging, older adults generally perform better than younger participants. Finally, we tested word fluency using a categorical fluency test (Cardebat et al., 1990), which assesses the following: integrity of lexico-semantic processes; strategic processes for searching and retrieval; and the integrity of phonetic and articulatory processes. Cognitive scores and significant differences between groups are reported in Table 2. Based on these scores, the OG was then subdivided into hOG and lOG subgroups (see “Results” section, Table 3).

**Table 2:** Mean (Mean_MAG; Mean_OG) and standard deviation (SD_MAG; SD_OG) of scores obtained on cognitive tests in each group and statistical values (*F, p*) for the inter-group comparisons.

	TMT-A	TMT-B	Digit span	FAB	Vocabulary	Fluency	Automatisms	PN	PPT
Mean—MAG	32.31	59.37	9.87	17.37	38.43	28.56	29.31	872.69	1558.40
Mean—OG	46.42	98.85	8.85	16.28	38.92	20.35	34.35	907.16	2169.20
SD—MAG	9.97	19.7	2.41	0.71	3.96	10.49	5.6	140.41	287.29
SD—OG	12.24	22.76	2.17	1.97	3.91	7.001	2.49	100.35	322.21
*F* value	−9.50	−23.74	0.64	2.49	0.275	6.62	10.97	0.814	−51.83
*p* value	0.005*	<0.05*	0.428	0.126	0.604	0.016*	0.003*	0.452	<0.05*

*MAG, Middle-aged group; OG, Older group; Trail Making Test A (TMT-A) and B (TMT-B); Digit span Memory Test; Frontal Assessment Battery (FAB); Vocabulary scale Mill-Hill; Categorical Fluency; Verbal Automatisms test; Picture Naming (PN); Pyramid Palm Tree Test (PPT). Bold values with an asterisk indicate significant differences*.

**Table 3:** Cerebral regions exhibiting an effect of age on the gray matter (GM) by comparing (a) middle-aged (MAG) > older (OG) adults; and (b) older (OG) > middle-aged (MAG) adults using two-sample *t*-tests.

Brain regions (Gray matter age effect)		H	BA	*k (mm^3^)*	*x*	*y*	*z*	*t*
[MAG > OG]	Inferior frontal gyrus	R	45	137	46	30	7	7.88
	Putamen	L	-	1952	−26	10	9	7.80
	[Insula]	L	13	-	−36	12	4	7.13
	[Inferior frontal gyrus] orb.	L	47	-	−37	22	0	6.80
	[Inferior frontal gyrus] triang.	L	45	-	−38	29	4	6.22
	Cerebellum	R	-	754	21	−68	−29	7.66
	Inferior frontal gyrus	R	45	401	38	30	4	7.62
	Supramarginal gyrus	L	40	611	−50	−33	28	7.14
	[Superior temporal gyrus]	L	22	-	−50	−36	20	6.85
	Superior occipital gyrus	L	18	248	−15	−84	28	6.96
	Angular gyrus	R	40	315	50	−42	41	6.89
	Middle frontal gyrus	R	10	62	22	51	9	6.68
	Postcentral gyrus	L	4	188	−55	−6	33	6.62
	Superior occipital gyrus	R	18	156	18	−91	27	6.58
	Supramarginal gyrus	R	40	397	45	−28	16	6.43
	[Transverse temporal gyrus]	R	41	-	43	−16	10	6.20
	[Middle temporal gyrus]	R	41	-	39	−24	14	5.96
	Middle frontal gyrus	L	10	52	−30	50	−3	6.24
	Cerebellum	L	-	34	−25	−66	−29	6.11
	Insula	R	13	21	45	−2	0	6.00
[OG > MAG]	*No significant result*						

*The statistical threshold for cluster and individual voxel was defined as p < 0.05 family-wise error (FWE) corrected for multiple comparisons (T > 5.77) with an extent voxel threshold defined as 20 voxels (voxel size = 1 mm^3^). Only regions revealing significant differences between groups were included. For each cluster, the region showing the maximum t-value was listed first, followed by the other regions in the cluster [in square brackets]. Talairach coordinates (x, y, z) of the peak and volume (k, mm^3^) of clusters are also shown. H, hemisphere; R, right hemisphere; L, left hemisphere; BA, Brodmann area*.

### MRI Acquisition

Magnetic resonance images (MRI) were acquired using a whole-body 3T Achieva Philips scanner (Philips Medical Systems, Netherlands) with a 32-channel head coil at the MRI facility IRMaGe in France. We acquired a T1-weighted high-resolution three-dimensional anatomical volume, by using a 3D Modified Driven Equilibrium Fourier Transform (MDEFT) sequence (number of slices = 160, echo time (TE) = 3.98 ms, repetition time (TR) = 25 ms, flip angle = 15°, field of view (FOV) = 256 × 240 × 160 mm, acquisition resolution 0.94 × 0.96 × 1.00 mm, acquisition matrix 272 × 249, reconstruction matrix 288 × 288, resolution reconstruction 0.89 × 0.83 × 1). None of the participants exhibited abnormalities in brain structures.

### Data Processing

#### Cognitive Scores

The effect of age on cognitive scores was determined by performing a MANOVA analysis on the performance obtained for each test in MAG and in OG controlled for gender and socio-educational effect (see “Results” section, Table 2). Cognitive scores were normalized based on the mean and SD considering all participants. In order to evaluate how cognitive performance varies among older adults and classify OG participants in the hOG and lOG, we first evaluated the normal distribution (Kolmogorov-Smirnov) of the scores for each test. We controlled for outliers by examining that scores did not exceed three times the interquartile interval.

#### MRI Data

##### Whole Brain Analyses

Data processing was performed using SPM12 release 6685 (Wellcome Department of Imaging Neuroscience, London, UK^1^) implemented in MATLAB 7 (Mathworks Inc., Natick, MA, USA). We processed the data via Diffeomorphic Anatomical Registration, using the Exponential Lie algebra algorithm (DARTEL, Ashburner and Friston, 2005; Ashburner, 2007) for the segmentation and normalization steps. The DARTEL segmentation procedure makes use of a number of tissue probability maps including GM, white matter (WM), CSF, soft tissue, skull and non-brain regions of the image. After segmentation, we performed a visual inspection and a quality check of the data, by applying the modules “display one slice for all images” and “check sample homogeneity using covariance” implemented in the VBM12 toolbox^2^. Next, the GM, WM and CSF tissue classes obtained during the segmentation step, were used to create a custom template based on our sample. For each participant, flow-fields were computed during template creation to provide the transformation matrix from each native image to the template. Finally, images obtained in the previous step were normalized to the MNI space (voxel size of 1 mm isotropic), modulated and smoothed using an 8-mm full width at half maximum (FWHM) Gaussian kernel. Importantly for subsequent statistical analyses, the total intracranial volume (TICV) was computed from the GM, WM and CSF modulated images. Morphological analyses were performed with the general linear model (Friston et al., 1995) with SPM12 implemented in MATLAB 7. We performed a whole brain analysis using the GM images in a two-sample *t*-test to compare GM volume between MAG and OG. We used an absolute implicit mask with a recommended threshold value fixed at *p* > 0.2 for GM voxel analyses (Callaert et al., 2014). In addition, in order to prevent potential bias related to brain size and gender differences between groups, the TICV and gender were included in the statistical model, as covariates of no interest. Differences in GM volume were considered significant if they exceeded a voxel-wise threshold of *p* < 0.05 family-wise error (FWE) corrected, with a minimum cluster extent of 20 voxels. Finally, we converted the MNI coordinates of voxels with maximal statistical significances into Talairach (Talairach and Tournoux, 1988) coordinates, by using the MNi2TAL function Matthew Brett^3^ to facilitate comparisons with other studies.

##### Cerebellum Analyses

Based on results reported by previous studies showing the role of the cerebellum in several cognitive domains (Buckner, 2013) and to deal with the poor alignment performance for cerebellar structures during spatial processing steps of the whole-brain analysis, we performed a specific cerebellum GM voxel based analysis using the SPM toolbox SUIT (Diedrichsen, 2006; Diedrichsen et al., 2009; Spatially Unbiased Atlas Template^4^). This cerebellar-specific pipeline also allowed us to better remove supra-tentorial GM which could bias final results. First, for each participant, the anatomical scans were manually reoriented in order to set them to the origin (0, 0, 0; anterior commissure). Next, the Isolate function of SUIT was used to obtain segmentation maps of the cerebellum. Then, GM isolated maps were corrected to exclude images with the GM located outside the cerebellum. Finally, the images were normalized to the SUIT space using the new DARTEL flow fields obtained from the SUIT toolbox, modulated and smoothed, using a 6 mm FWHM Gaussian kernel.

## Results

### Cognitive Scores

#### Aging Effect

As shown in Table 2, and relative to the OG, the MAG were faster for TMT-A (visual scanning and processing speed; *F*_(1,30)_ = −9.5 *p* = 0.005) and PPT (semantic processing; *F*_(1,30)_ = −51.834, *p* < 0.05). The OG also produced more words than the MAG for categorical fluency (*F*_(1,30)_ = 6.624, *p* = 0.016). For TMT-B (mental flexibility) the OG had lower scores than the MAG (TMT-B, *F*_(1,30)_ = 23.737, *p* < 0.05). We did not observed difference between MAG and OG on the FAB (global frontal functioning; *F*_(1,30)_ = 2.494, *p* = 0.126) and on the PN (*F*_(1,30)_ = 0.814, *p* = 0.375). As expected, the OG had higher scores for verbal automatisms (*F*_(1,30)_ = 10.975, *p* = 0.003) than the MAG. The cognitive-test results suggest that aging is mainly associated with lower performances for domain-general processes—and, to a lesser extent, for domain-specific processes.

### MRI Morphological Results

#### Whole Brain

**Figure 1:**
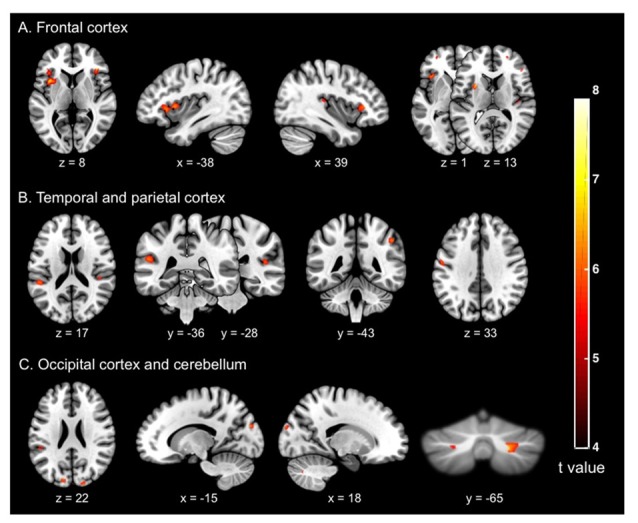
Cerebral regions (whole brain analysis) showing the significant differences between groups (older adults vs. middle-aged adults) on the gray matter (GM) volume (in mm^3^) of **(A)** the frontal cortex; **(B)** the temporal and parietal cortices; and **(C)** the occipital cortex and cerebellum. The significance threshold for clusters and individual voxel level was defined as *p* < 0.05 family-wise error (FWE), corrected for multiple comparisons (*T* > 5.77), with extent threshold defined as 20 voxels (voxel size = 1 mm^3^).

The whole brain voxel-based GM analyses and comparisons between groups revealed significantly smaller GM volume for OG compared to MAG in frontal, parietal, temporal and occipital regions, as well as of the cerebellum (Table 3 and Figure 1). More specifically, we observed bilateral GM decreased of the middle (BA 10) and inferior (BA 45, 47) frontal gyri, insula, supramarginal (BA 40), superior occipital (BA 18), cerebellum, as well as of the right angular (BA 39), superior temporal (BA 41), left postero-superior temporal (BA 22) and post-central (BA 3, 2, 1) gyri.

#### Cerebellum

**Figure 2:**
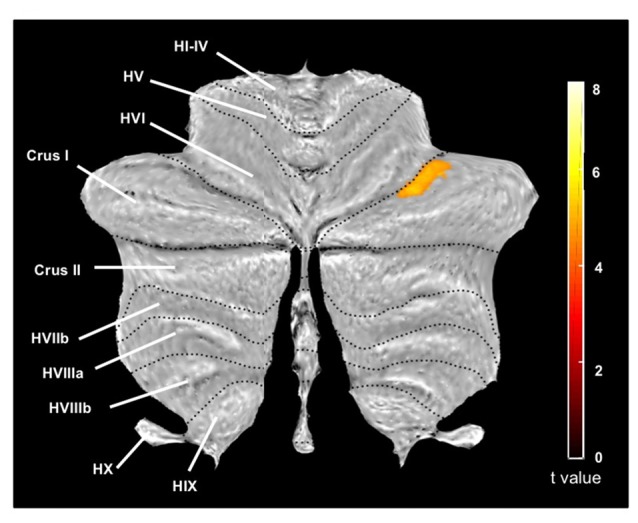
Cerebellum region (cerebellar analysis) showing significant differences between groups (older adults vs. middle-aged adults) on the GM (in mm^3^), and represented on a two-dimensional template with lobules indicated by roman numerals from I to X with a prepended H and V for the hemispheric and vernal compartment, respectively. On this image, the largest lobule HVII is divided into Crus I, Crus II (both corresponding to HVIIa) and HVIIb. Statistical significance threshold for the cluster level was set at *p* < 0.05 FWE corrected for multiple comparisons. We reported only one significant cluster (*T* = 4.99) belonging to the Crus I of the right cerebellum, with extent threshold defined as 20 voxels (voxel size = 1 mm^3^).

**Figure 3:**
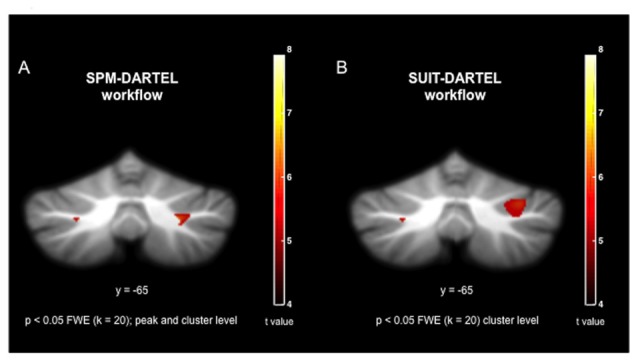
Visual comparison of co-registration during spatial processing using **(A)** the SPM-DARTEL pipeline; and **(B)** the SUIT-DARTEL pipeline.

The cerebellum voxel-wise GM analysis using the SUIT toolbox (Diedrichsen, 2006; Diedrichsen et al., 2009) revealed a significant cluster (340 mm^3^) in the right cerebellar hemisphere (*p* < 0.05 FWE corrected) reflecting decreased GM volume in the OG. The location of this region corresponds to the lobule crus I of the right cerebellar hemisphere (Figure 2). This result was displayed on a two-dimensional cerebellum template implemented in the SUIT toolbox (Diedrichsen and Zotow, 2015). Figure 3 shows a comparison of the two spatial processing pipelines used.

## Discussion

The present study aimed to assess: (1) how aging impacts GM volume; in relation to; (2) how aging impacts performance in tasks involving both domain-general (i.e., executive functioning) and domain-specific functions (i.e., language and semantic memory).

The older adults included in this study exhibited: (a) decreased performance on tests reflecting executive functioning, frontal efficiency and processing speed; and (b) difficulty in retrieving and generating words and performing semantic processing. Older adults also showed a greater frequency of verbal automatisms. These findings are consistent with previous studies (Bherer et al., 2004; Zelazo et al., 2004; Huizinga et al., 2006; Collette and Salmon, 2014; Baciu et al., 2016; Boudiaf et al., 2016; Hoyau et al., 2017) showing a generally lower cognitive performance in the course of normal aging.

At the cerebral level, VBM analyses comparing the OG to the MAG revealed that aging was also associated with significantly smaller bilateral GM volume in frontal, temporal, parietal and occipital regions. Smaller GM volume in the frontal lobe concerned first the middle frontal gyrus, with a greater extent in the right hemisphere. This region is known to be involved in cognitive control and to play a key role in reorienting attention according to the task demands (Rossi et al., 2009; Japee et al., 2015). This pattern is consistent with a number of studies reporting changes in the capacity to switch between tasks or to disregard distractors in older participants (Rajah et al., 2008, 2010). GM volume differences also concerned the bilateral inferior frontal gyrus, as well as the left superior temporal gyrus. These regions are involved in motor planning and articulation, and in executive processing related to accessing phonological representations (Hickok, 2009). Finally, significantly smaller GM volume of the bilateral insula, involved in word retrieval and generation (Abel et al., 2009) was observed in OG as compared to MAG, with an extensive cluster in the left hemisphere. Interestingly, the OG in the present study exhibited lower cognitive performance for a number of language tests reflecting lexical retrieval and generation and semantic processing, and recruiting executive functions such as switching, inhibition and strategic search abilities, as well as working memory processes (Troyer et al., 1997; Bryan and Luszcz, 2000). Together with behavioral results, the smaller GM volume of frontal areas in aging observed here therefore appears to be consistent with previous studies suggesting a relationship between executive decline and GM volume of the frontal lobe (Good et al., 2001; Tisserand et al., 2004; Lemaitre et al., 2012; Manard et al., 2016).

The age-related smaller GM volume observed in our study also pertained to regions that are part of a larger fronto-parietal network involved in attentional processes, including the right supramarginal and angular gyri. These regions are involved in attention processes related to executive functions (Seghier, 2012) and the control of attentional shifts in space (Chen et al., 2012) and exert top-down attention-switching control signals to visual areas (Rossi et al., 2009). This network, including notably the left frontal cortex as a hub, in relation to the dorsal attentional network (DAN) and the default-mode-network (DMN), have been also reported to play a key role to maintain the memory performance in normal and pathological aging (Franzmeier et al., 2017a,b). Finally, diminished GM volume of the bilateral superior occipital gyri and transverse temporal gyrus (primary auditory cortex) was also observed in the OG. This result is consistent with a previous finding that reported global thinning of the cerebral cortex, including the cerebral regions dedicated to perceptual processing with age (Salat et al., 2004). To sum up, our findings of age-related smaller GM volume in frontal, temporal and occipital cortical areas consistent with previous results, which suggest that decreased GM volume of these areas is associated with diminished executive efficiency (Tisserand et al., 2004). Although previous structural and functional studies on cognitive and GM changes in normal aging mostly focused on the cerebral cortex, the present study also addressed GM changes in the cerebellum, by using a specific analysis pipeline optimized for this structure. VBM analyses in the cerebellum revealed significantly smaller GM volume in the OG in the right lobule HVIIa, including the crus I and crus II regions (*x* = −36; *y* = 12; *z* = 4; *t*-value = 7.13). Over the past decades, evidence has indicated that cerebellar functioning extends beyond the scope of classical sensorimotor control and is related to domains such as attention, language, executive function and social cognition. In a recent opinion article, Sokolov et al. (2017) assert that cerebellar computations—based on of their extensive reciprocal connections with the frontal, parietal and temporal associative cortices—are universal across sensorimotor and associative processes, along with two key phenomena, prediction and error-based learning. Recent results show that the most human cerebellum maps are related to cerebral association networks. Furthermore, Kelly and Strick (2003) found that large regions near crus I and crus II exhibit connections with prefrontal cortex area 46 (which is involved in executive processes) in non-human primates. Importantly, this cortical circuit between the cerebellum and prefrontal cortex overlaps with regions dedicated to motor control (Buckner, 2013). Another study revealed that lobule HVIIa, including crus I, is connected to a large association network involved in executive control (Habas et al., 2009). The cerebellum is also significantly involved in language, social cognition and cognition in general (Buckner, 2013). Right crus I atrophy in older adults may be related to their lower semantic cognitive scores, given that this region is connected to the left cerebral regions that are thought to be involved in the semantic demands of task processing (Petersen et al., 1989; Stoodley, 2012). Overall, the smaller GM volume in the cerebellum observed in the OG extends previous findings on GM atrophy in normal aging related to cognitive abilities (Lemaître et al., 2005; Raz et al., 2005; Driscoll et al., 2009) and particularly to executive functions.

The present VBM study has a number of limitations. In particular, as this analysis is exclusively cross-sectional it provides no information concerning causality and temporal changes. This issue remains particularly relevant for gaining a greater understanding of the concurrent changes in cognitive scores and GM volume as they relate to cognitive performance. Indeed, one critical question remains regarding the extent to which smaller GM volume in older relative to middle-aged participant also varies according to cognitive performance. It is for example possible that older adults with higher cognitive performance exhibit less GM volume reduction than those with lower cognitive performance. For example, a recent study adopted a cross-sectional approach to structural MRI to investigate differences in GM volume in relation to cognitive performance (Nissim et al., 2017). Using a longitudinal approach, another study (Tisserand et al., 2004) carried out a 3-year comparison of GM density between a “stable” and a “decliner” group. These studies suggested a link between GM volume of the frontal lobe and cognitive performance, suggesting that GM volume in this region might be a predictor of cognitive functioning in older adults. When it comes to normal or pathological aging, cerebral reserve (Katzman, 1993; Satz, 1993) and cognitive reserve (Stern, 2002, 2009) are key concepts that need to be taken into consideration. Cerebral reserve designates the amount of cerebral deterioration that can be tolerated before a critical threshold is reached whose clinical or functional consequences are inevitable, whereas cognitive reserve refers to the ability to use the available cerebral reserve to perform a task flexibly and efficiently. With respect to this conceptual framework, several researches on animal and humans posit the benefic role for successful aging of a rich environment and a greater education to maintain cognitive performance relatively well in face of age-related brain modifications and pathology (Freret et al., 2015; Franzmeier et al., 2017a,b; Gelfo et al., 2018). However, due to the relatively small sample of older participant, further analyses comparing GM volume between low- and high performers could not be performed in the present study. Moreover, our sample size remains too week to discern subtle GM differences or a distinct pattern of results depending on the sex of the participants (Weiner et al., 2017). Further studies including a larger sample would thus allow to precisely test these hypotheses. Further research, including the collection of genetic, structural and functional brain connectivity information, is also needed on the individual trajectories of the brain structure in normal aging, and in relation to cognitive scores. Such research would help to make the distinction between transitional phases and normal cognitive and brain aging patterns in pathological settings.

## Conclusion

The present VBM study aimed to assess the effect of aging on GM and to interpret results in relation to the cognitive performance. Overall, this study provided a broad picture on domain-general cognitive functioning, but also language-specific processes in normal aging, as well as the associated anatomical differences in the whole brain, including the cerebellum. A decrease of GM volume was observed in several regions in older compared to middle-aged adults, interpreted in relation to lower cognitive scores in tests assessing either domain general or specific processes. These results replicate and further support previous findings suggesting a general decline in cognitive processes associated with smaller GM volume in the course of normal aging. Moreover, these results emphasize the importance of taking into account the cerebellum structure when studying changes associated with normal aging, which clearly extend beyond the cerebral cortex. These findings open up new perspectives for the development and application of innovative training methods and programs that aim to promote successful normal aging.

## Author Contributions

MB and NB: conceived the study. NB, CP, AK and AJ: data acquisition. SR, EH and FR: data processing. SR, EH, MB and LK: manuscript writing.

## Conflict of Interest Statement

The authors declare that the research was conducted in the absence of any commercial or financial relationships that could be construed as a potential conflict of interest.

## Footnotes

^1^www.fil.ion.ucl.ac.uk/spm/

^2^http://www.neuro.uni-jena.de

^3^http://www.mrc-cbu.cam.ac.uk/Imaging

^4^http://www.diedrichsenlab.org/imaging/suit.htm
